# The general health status of employees in Germany aged 45 to 59 years (Ü45-Check) – a cross-sectional study

**DOI:** 10.1186/s12889-025-24778-7

**Published:** 2025-09-24

**Authors:** Linda Kalski, Tilman J. Pulst Caliman, Franziska Greiß, Athanasios Karathanos, Lorena Hafermann, Charleen Pächter, Laura Völkel, Carolin Herrmann, Maja A. Hofmann, Bernd Wolfarth

**Affiliations:** 1 Institute of Sport Science, Humboldt-Universität zu Berlin, Berlin, Germany; 2 Department of Sports Medicine, Charité – Universitätsmedizin Berlin, Berlin, Germany; 3 Institute of Biometry and Clinical Epidemiology, Charité – Universitätsmedizin Berlin, Corporate Member of Freie Universität Berlin and Humboldt-Universität zu Berlin, Berlin, Germany; 4 Mathematical Institute, Heinrich Heine University Düsseldorf, Düsseldorf, Germany; 5 Department of dermatology and venereology, Charité – Universitätsmedizin Berlin, Berlin, Germany; 6 Federal German Pension Insurance Berlin-Brandenburg, Berlin, Germany

**Keywords:** Screening, Prevention, Health status, Occupational groups, ISCO-08

## Abstract

**Objective:**

This study was performed to evaluate the general health status of employees aged 45–59 years using data from the Ü45-Check study (2021–2024). In addition, the health status of individual occupational groups was assessed to investigate potential associations within these groups.

**Methods:**

Clinical and occupational data were collected from 1,040 employees aged 45–59 years from Berlin and Brandenburg, Germany, who participated in the Ü45-Check study. The data were derived from a preventive health examination conducted to identify potential preventive or rehabilitation needs. The clinical examination included anthropometric measurements, bioelectrical impedance analysis, handgrip strength, 12-lead resting electrocardiogram, systolic and diastolic blood pressure, anamnesis, and blood analyses. To categorize health status by occupation, the International Standard Classification of Occupations 2008 (ISCO-08) was applied.

**Results:**

Data from 1,040 participants (*n* = 631 male, 61%) revealed occupational and sex-related disparities. The mean body mass index (BMI) of the total sample was 26.89 kg/m^2^ (sd = 4.90). *Plant and Machine Operators and Assemblers* had a mean BMI of 28.25 kg/m^2^ (sd = 5.19), while those in *Elementary Occupations* had a mean BMI of 28.45 kg/m^2^ (sd = 4.88), which was the highest among the occupational groups. In terms of percent body fat, the highest means were found in *Elementary Occupations*, 31.03% (sd = 8.65), and *Service and Sales Workers*, 32.35% (sd = 8.73). Mean systolic blood pressure values were also elevated: *Elementary Occupations* (139.02 mmHg, sd = 15.92), *Skilled Agricultural*,* Forestry*,* and Fishery Workers and Craft and Related Trades Workers* (139.63 mmHg, sd = 15.60), and *Plant and Machine Operators and Assemblers* (139.78 mmHg, sd = 17.90). Resting electrocardiograms and clinical examinations were largely unremarkable. However, blood analyses revealed elevated total cholesterol (mean = 204.03 mg/dL, sd = 40.18) and low-density lipoprotein (mean = 126.28 mg/dL, sd = 35.30) in both women and men, exceeding reference ranges.

**Conclusion:**

These results highlight occupational and sex-based disparities in anthropometric and clinical measures, pointing to significant health concerns such as obesity and increased cardiovascular risk. The findings underscore the importance of occupational health initiatives tailored to specific job demands and sex differences.

**Clinical trial registration:**

DRKS ID: DRKS00030982.

**Supplementary Information:**

The online version contains supplementary material available at 10.1186/s12889-025-24778-7.

## Introduction

It is well known that industrialized countries are facing steadily aging populations and declining birth rates. In Germany, these demographic trends are placing increasing strain on health and pension insurance systems [1]. Early retirement remains a significant phenomenon: One analysis found that more than 40% of those receiving disability pensions had not used any medical rehabilitation services within the 5 years prior to retirement [2]. At the same time, many countries, including Germany, are raising the retirement age (in Germany, gradually to 67 years by 2029) to help sustain social security funding [3]. When the entire cohort of the post-war high birth rate generation (born 1954–1969, commonly referred to as the “Baby Boomer”) retires by 2036, a substantial workforce will be lost from the German and European labor markets. By 2023, 1.8 million Baby Boomers (born 1954–1957) had already taken early retirement, corresponding to approximately 44% of each birth cohort. If current retirement regulations remain unchanged, from 2025 onwards, roughly one million Baby Boomers per year will begin receiving statutory old-age pensions before reaching the standard retirement age [4]. Maintaining the health and work ability of subsequent generations (e.g., Generation X) will therefore be essential to keep workers in the labor market for as long as possible and to counteract the challenges posed to the health and social insurance systems [4, 5]. The goal is not only to stabilize social insurance systems but also to address the growing shortage of skilled workers [5].

The risk of developing chronic diseases, and consequently receiving a disability pension, can be increased by various factors such as smoking, obesity, musculoskeletal symptoms, mental health issues, or respiratory diseases [6]. Obesity is one of the most significant health risk factors, raising the likelihood of developing diabetes, cardiovascular diseases, or hypertension. It can also lead to a substantial decline in quality of life and, as a result, impact work ability [7–9]. In 2020, Bergman et al. [8] found that both good sleep quality and a low body mass index (BMI) were positively associated with quality of life and self-rated work ability, whereas occupational stress, anxiety, depression, and illness were negatively associated.

The level of occupational physical activity (sedentary, moderate, or vigorous), which characterizes most people’s daily lives, appears to influence the risk of developing diseases, as do leisure-time physical activity and dietary habits [10–12]. Strenuous occupational activity can negatively affect health, leading to musculoskeletal disorders, reduced work capacity, and sick leave due to work-related injuries [10]. Employees in agriculture or manual occupations have the highest chance of occupational injuries [13]. Similarly, sedentary behavior is increasingly recognized as a risk factor for higher mortality and the development of chronic diseases [11]. This concern is underscored by the annual health report of the National Health Insurance, which states that the amount of time the German population spends sitting has risen steadily by 1.5 h per year over the past 7 years, reaching an average of 9.2 h per working day in 2023 [12]. Across Europe as a whole, sitting time in the age range of most of the working population (21–65 years) increased significantly between 2013 and 2022 [14].

International studies also report consistent associations between occupational activity patterns and cardiometabolic health indicators such as BMI, blood pressure, and lipid profiles; however, most evidence is based on self-reported data and does not apply harmonized occupational classifications, limiting cross-country comparability [15, 16].

The costs to the government are significant because of the rise in disability pensions and chronic diseases. Therefore, from an economic standpoint, the goal should be to maintain employees’ ability to work and to counteract the increase in chronic illnesses through preventive measures [17]. Preventive health check-ups to identify the need for prevention or rehabilitation represent an important step in this direction [18]. However, maintaining work ability is not only beneficial economically but also on an individual level, as the ability to work positively influences quality of life, which in turn enhances overall well-being [8]. The principle of “prevention before rehabilitation before retirement” largely shapes the work of the German Pension Fund (Deutsche Rentenversicherung) [2] and could be significantly strengthened by introducing nationwide preventive health check-ups. Early preventive measures could benefit individuals while also offering a sustainable response to the challenges posed by demographic change [8, 18].

In this context, general health status is a key concept for assessing prevention and rehabilitation needs in the working population. Despite its relevance, no recent study has comprehensively assessed this status in midlife employees (aged 45–59 years) residing in an urban environment in Germany, using both objective clinical measures and self-reported health information. Understanding the current health situation of Generation X (born 1962–1980), the successors of the Baby Boomer generation, can inform targeted, occupation-specific prevention strategies aimed at maintaining work ability and reducing the long-term burden of chronic disease and disability pensions.

There is a lack of recent, objectively measured, occupation-stratified health profiles from Germany that can be compared to international data. In Germany, sector-specific disparities have been observed in early-onset Type 2 diabetes [19], and strenuous care work is linked to adverse self-rated health [20]. Although the International Standard Classification of Occupations 2008 (ISCO-08) is an established classification system enabling international comparability of labor data [21–23], few studies have provided objectively measured clinical health profiles stratified across ISCO-08 major groups, particularly for midlife employees. While recent German research has examined associations between occupation (coded by ISCO-08) and coronary heart disease [24], such analyses rely on disease outcomes rather than comprehensive clinical assessments like BMI, body composition, blood pressure, lipid panels, or functional strength.

In this study, health status refers broadly to an individual’s overall physical and functional condition, assessed using both objective clinical measures and self-reported health information. General health status, as used here, is a composite classification derived from these indicators. A favorable general health status is defined as having values within the reference ranges for all objective measures and reporting no relevant functional or health limitations in the anamnesis; an unfavorable general health status refers to values outside reference ranges and/or self-reported health problems or limitations.

To determine the need for prevention or rehabilitation among employees aged 45–59 years, each participant underwent a medical examination and completed a questionnaire (Ü45-Screening) [25–27]. Both tools were designed to assess overall health status. The primary research question of the Ü45-Check study was to compare the outcomes of the medical examination with the questionnaire results in identifying prevention and rehabilitation needs. Additionally, we evaluated which medical parameters most strongly influenced the determination of prevention or rehabilitation needs. We also examined the relationship between the questionnaire, the medical examination, and the Risk Index – Disability Pension [28]. The findings related to these questions are the subject of a separate article [29]. The extensive cross-sectional data collected allow for further in-depth analysis. In the present analysis, we address the research gap by providing a clinically assessed, ISCO-stratified health profile of midlife employees in Germany and by examining differences across major occupational groups and between sexes. To this end, we combine objectively measured clinical health indicators (anthropometrics, blood pressure, lipid panels, handgrip strength) collected by trained physicians with standardized self-reported health information. The secondary objectives of this study included evaluating the general health status of participants as well as the health status of specific occupational groups.

Study questions

1. What is the general health status of a sample of German employees aged 45–59 years?
2. What is the general health status of specific occupational groups within that age range?

## Methods

### Study design and population

The Ü45-Check was conducted as a prospective cross-sectional study to assess the need for prevention and rehabilitation through preventive health examinations and a questionnaire survey among employees aged 45–59 years who were insured with the German Pension Fund (Deutsche Rentenversicherung, DRV) and invited directly by the German Pension Fund to participate. The screenings targeted employees aged 45–59 years living in Berlin and the federal state of Brandenburg, Germany. Participants were randomly selected by the German Pension Fund and invited to take part in the Ü45-Check over 4 consecutive calendar years, from 2021 to 2024. Full details of the study design have been published elsewhere [25].

## Setting

The Ü45-Check was conducted as a preventive health examination at the outpatient clinic of the Department of Sports Medicine at Charité – Universitätsmedizin Berlin/Humboldt-Universität zu Berlin, Germany. The examination included both clinical evaluations and a questionnaire survey, targeting employees aged 45–59 years from the Berlin and Brandenburg regions. The exclusion criteria were individuals residing outside of Berlin or Brandenburg, those lacking proficiency in German or English, individuals already in early retirement, and those who had recently undergone rehabilitation. In Germany, certain groups are not insured by the German Pension Fund, such as self-employed individuals, civil servants and judges, lawyers, soldiers, farmers, and doctors, and were therefore also excluded from the study.

Following the Ü45-Check, participants promptly received personalized health status results and took part in a consultation with a physician, who provided a detailed overview of the findings.

### Preventive health examination

The comprehensive preventive health examination lasted 120 min and included a range of assessments, such as anthropometric measurements (height, weight, hip and waist circumference, and bioelectrical impedance analysis), handgrip strength, a 12-lead resting electrocardiogram, systolic and diastolic blood pressure (SBP and DBP), anamnesis, and blood sampling. Handgrip strength was assessed using a calibrated dynamometer and is considered a simple, reliable indicator of overall muscle strength and functional capacity, making it a widely used marker in both clinical and epidemiological research [30, 31].

.A detailed breakdown of all parameters is available in the study design publication [25]. The physical examination primarily focused on evaluating the cardiovascular system, lungs, and abdomen. Blood samples were collected under fasting conditions.

During the subsequent physician consultation, a thorough medical history was taken with attention to relevant details. Current health concerns and medical needs were addressed, and recommendations for further treatment or assessment were provided. The consultation also included discussions about regular physical activity, health habits, and the importance of a balanced diet.

### Classification of occupational groups

To examine the general health status of individual occupational groups, participants were classified into one of eight major occupational groups based on the job descriptions they provided in the questionnaire. The categorization followed the ISCO-08, a manual published by the International Labour Organization (ILO) that assigns occupational titles to job-specific tasks [23, 32]. The ISCO-08 system consists of 10 major groups, each comprising one or more sub-major groups, which are further divided into minor groups derived from unit groups. Each unit group includes several occupations that share similar skill levels and specializations. Every job title is assigned a four-digit ISCO-08 code, with each digit corresponding to a classification level (major, sub-major, minor, unit group). For this study, the analysis was limited to assigning job titles to their respective major groups. Accordingly, each job title was matched to a major group number. No participants were assigned to group 0 (Armed Forces Occupations) because individuals in military roles were not included in the selection process. Additionally, main groups 6 and 7 were combined because of the low number of participants in group 6 and the content-related similarity between the two groups. The final occupational groups used in this study were as follows:

- Managers
- Professionals
- Technicians and Associate Professionals
- Clerical Support Workers
- Service and Sales Workers
6. and Skilled Agricultural, Forestry, and Fishery Workers and Craft and Related Trades Workers
- Plant and Machine Operators and Assemblers
- Elementary Occupations
- Unemployed Persons

For completeness, individuals who reported being unemployed are displayed as a separate category (“Unemployed Persons”), although this group does not form part of the official ISCO-08 major groups. The classification of job titles into major groups was carried out independently by two individuals using the ISCO-08 system. Job titles were first searched using the search function on the ILO Department of Statistics (ILOSTAT) website. If no direct match was found, classification was based on the Excel tables provided by ILOSTAT, which outline the main groups along with their associated skill levels and job descriptions [23, 32].

### Statistical analysis

**Fig. 1:**
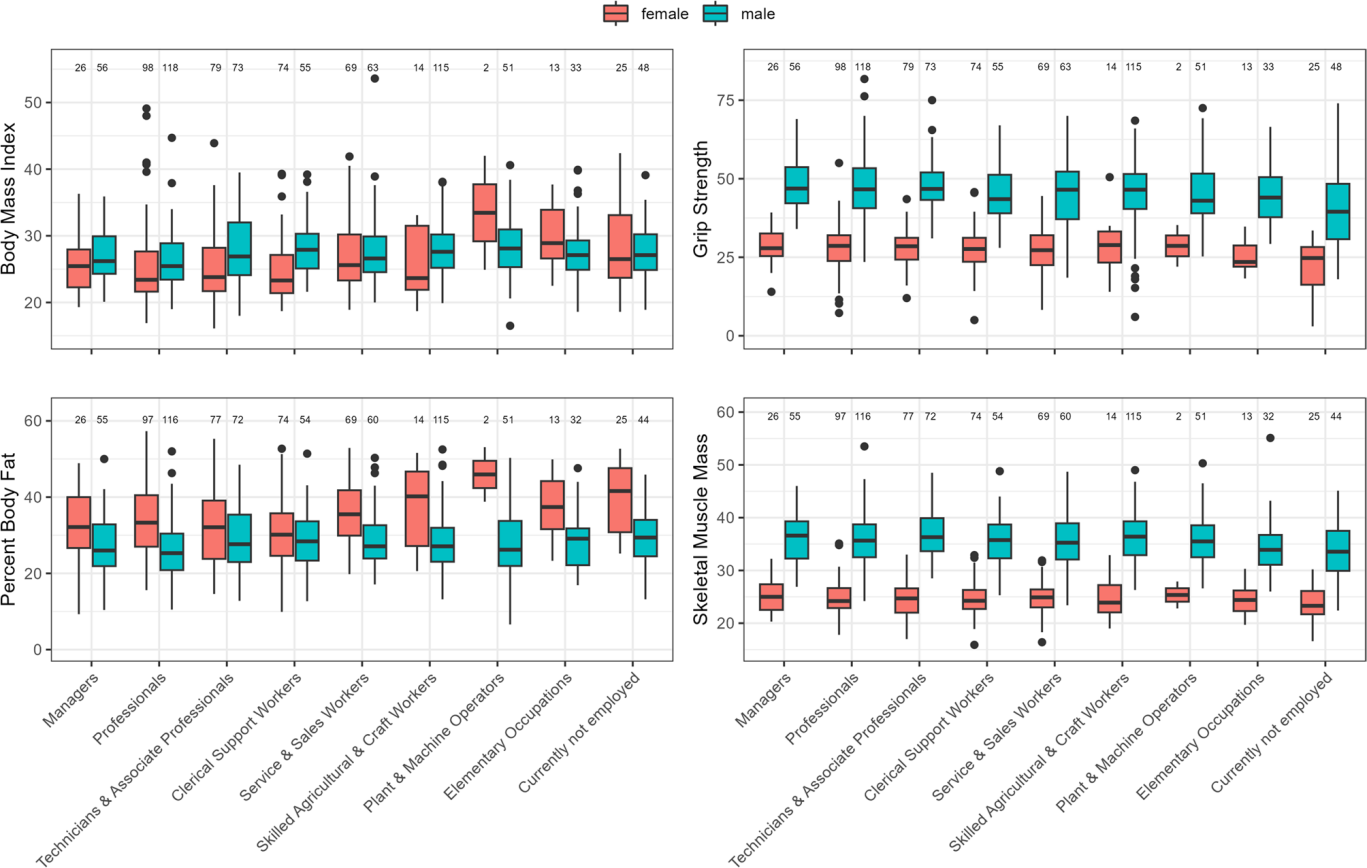
Boxplots showing the distribution of key anthropometric and physical variables (BMI, PBF, handgrip strength, and SMM) across different occupational groups, stratified by sex. Red boxes represent female participants, and blue boxes represent male participants. The numbers above each boxplot indicate the sample size for the respective subgroup

**Fig. 2:**
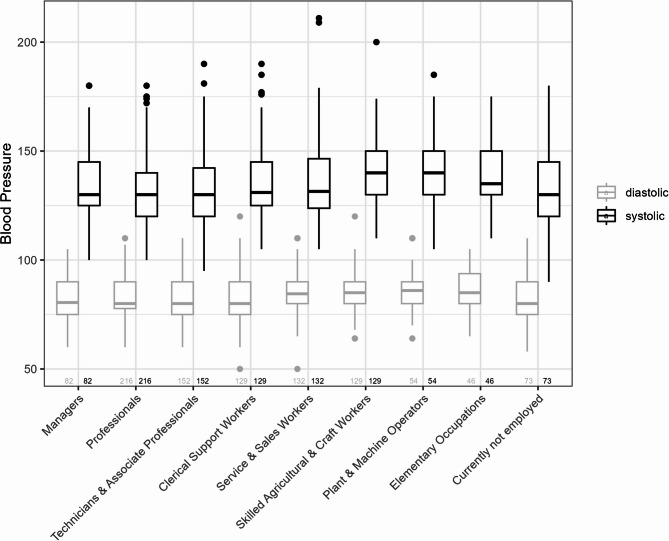
Boxplots of systolic and diastolic blood pressure across different occupational groups. The numbers below each boxplot indicate the sample size of the respective subgroup

Because this study analyzed secondary endpoints of the Ü45-Check study, all analyses were exploratory in nature. First, all demographic characteristics and occupations are described descriptively, stratified by sex. Means and standard deviations (sd) are presented for age and BMI, while categorical variables are reported using absolute and relative frequencies (Table 1). Results for BMI, percent body fat (PBF), handgrip strength, and skeletal muscle mass (SMM) by occupation and sex are shown using boxplots (Fig. 1). Boxplots are also provided for SBP and DBP by occupation (Fig. 2). Results from the resting electrocardiogram and clinical examination are presented as absolute and relative frequencies. For the blood analysis, values were normalized to specific thresholds to enable comparability. The normalization used the formula $\:{x}_{norm}=\:\frac{x-{t}_{low}}{{t}_{upp}-{t}_{low}}$, where *x* represents the individual blood measure and $\:\left[{t}_{low},\:{t}_{upp}\right]$ the sex-specific lower and upper thresholds. In this way, values at the lower threshold were assigned 0, and those at the upper threshold were assigned (1) Because some participants’ values fell significantly below or above these limits—leading to poor data representation—results are displayed only within the range of − 0.5 to (2) Values outside this range are noted on the left and right sides of the graph. Boxplots are presented for all blood measures. All analyses were performed using R software (version 4.3.1).

**Table 1:** Demographic characteristics

Parameter	Total (%)	Male (%)	Female (%)
*N*	1,040	631 (61%)	409 (39%)
Age, years			
* mean (sd)*	52.93 (4.17)	52.93 (4.21)	52.93 (4.12)
Residence			
* Berlin*	887 (85%)	532 (60%)	355 (40%)
* Brandenburg*	153 (15%)	99 (65%)	54 (35%)
BMI, kg/m^2^			
* mean (sd)*	26.89 (4.90)	27.45 (4.36)	26.02 (5.51)
ISCO-08			
1 *Managers*	82 (7.9%)	56 (68%)	26 (32%)
2 *Professionals*	216 (21%)	118 (55%)	98 (45%)
3 *Technicians and Associate Professionals*	152 (15%)	73 (48%)	79 (52%)
4 *Clerical Support Workers*	129 (12%)	55 (43%)	74 (57%)
5 *Service and Sales Workers*	132 (13%)	63 (48%)	69 (52%)
6 and 7 *Skilled Agricultural*,* Forestry*,* and Fishery Workers and Craft and Related Trades Workers*	129 (12%)	115 (89%)	14 (11%)
8 *Plant and Machine Operators*,* and Assemblers*	53 (5.1%)	51 (96%)	2 (3.8%)
9 *Elementary Occupations*	46 (4.4%)	33 (72%)	13 (28%)
10 *Unemployed Persons*	73 (7.0%)	48 (66%)	25 (34%)
* No Answer*	28 (2.7%)	19 (68%)	9 (32%)

*BMI* body mass index, sd standard deviation, *ISCO-08* International Standard Classification of Occupations 2008

### Ethics approval and consent to participate

Ethics approval was granted by the Ethics Committee of the Faculty of Culture, Social and Educational Sciences, Humboldt-Universität zu Berlin, on 20 August 2020 (reference number: HU-KSBF-EK_2020_0010). The study was conducted in accordance with the Declaration of Helsinki for research involving human subjects. All participants were informed by study staff about the study procedures, data storage, confidentiality, and the pseudonymization of their data. Written informed consent was obtained from all participants, allowing the study center to use the data for research analyses and publication.

### Trial registration

The trial was registered in the German Clinical Trials Register (DRKS-ID: DRKS00030982). It was retrospectively registered on 27 December 2022: https://drks.de/search/de/trial/DRKS00030982.

## Results

### Study characteristics

Data from 1,040 participants were analyzed. Table 1 presents a descriptive overview, including information on age, sex, place of residence (federal state), BMI, and classification according to ISCO-08 occupational groups.

Based on World Health Organization guidelines, 8 (0.8%) participants were underweight (BMI < 18.5 kg/m^2^), 407 (39.1%) had a normal BMI (18.5–24.9 kg/m^2^), 370 (35.6%) were classified as pre-obese (25.0–29.9 kg/m^2^), 197 (18.9%) had obesity class I (30.0–34.9 kg/m^2^), 45 (4.3%) had obesity class II (35.0–39.9 kg/m^2^), and 13 (1.3%) had obesity class III (≥ 40 kg/m^2^) [33]. The mean BMI for the total sample was 26.89 kg/m^2^ (sd = 4.90), with a mean of 27.45 kg/m^2^ (sd = 4.36) for men (*n* = 631) and 26.02 kg/m^2^ (sd = 5.51) for women (*n* = 409).

### Anthropometric data

Figure1 presents boxplots illustrating the distribution of selected anthropometric variables across different occupational groups, with results shown separately for male and female participants. The variables analyzed include BMI, PBF, handgrip strength, and SMM.

The boxplots for BMI indicate that certain occupational groups, particularly *Plant and Machine Operators and Assemblers* (male: median [interquartile range] = 28.1 kg/m^2^ [25.0, 30.7]; female: 33.5 kg/m^2^ [29.2, 37.7]) and those in *Elementary Occupations* (male: 27.1 kg/m^2^ [24.9, 29.3]; female: 28.9 kg/m^2^ [26.6, 33.9]), had higher median BMI values, suggesting a greater prevalence of overweight and obesity in these groups. These occupations also tended to show lower SMM values in the sample. Female participants (shown in red) in these occupational groups generally exhibited higher BMI values than their male counterparts (shown in blue). Among *Professionals*, men showed a higher median BMI (25.5 kg/m^2^ [23.4, 28.9]) than women in the same group (23.4 kg/m^2^ [21.6, 27.7].

Among males, the occupational groups with the highest median PBF were *Clerical Support Workers* (28.4 [23.2, 33.9]) and those in *Elementary Occupations* (29.1% [22.0, 32.0]). This suggests that male employees in clerical support roles tend to have a higher PBF than men in other occupational groups. Among females, the highest median PBF was observed in *Plant and Machine Operators and Assemblers*, at 46.0% (38.8, 53.1). However, this result should be interpreted with caution because only two female participants from this occupational group were included in the study. Nevertheless, female employees in *Skilled Agricultural*,* Forestry*,* and Fishery Workers and Craft and Related Trades Workers* also showed a notably high PBF, with a median of 40.2% (26.6, 47.2).

The handgrip strength data show that *Skilled Agricultural*,* Forestry*,* and Fishery Workers and Craft and Related Trades Workers* exhibited higher median handgrip strength values, particularly among females, at 28.9 kg (23.3, 33.2), which is plausibly related to the physically demanding nature of their jobs. Among men, the highest median values were observed in *Managers* 46.9 kg (42.2, 53.7), with similarly elevated scores in the SMM 36.6 kg (32.3, 39.3). Among women, *Managers* also displayed higher SMM values, 25.0 kg (22.5, 27.4), compared to other occupational groups.

SMM was also higher among *Skilled Agricultural*,* Forestry*,* and Fishery Workers and Craft and Related Trades Workers* (male: 36.4 kg [32.9, 39.3]). These occupations typically involve substantial physical activity, which contributes to greater muscle mass. This is supported by data on working posture and occupational physical activity levels: 90 (70%) of *Skilled Agricultural*,* Forestry*,* and Fishery Workers and Craft and Related Trades Workers*, 35 (76%) of those in *Elementary Occupations*, and 13 (24%) of *Plant and Machine Operators and Assemblers* reported performing heavy physical labor. By contrast, more than 55% (*n* = 579) of participants from the first four occupational groups reported predominantly sedentary work with low physical activity. Among *Clerical Support Workers*, 105 (81%), and among *Managers*, 64 (78%) reported working primarily in sedentary roles.

### Blood pressure

Figure2 presents boxplots illustrating the distribution of SBP and DBP across different occupational groups.

There were visible differences between occupational groups in terms of median SBP and DBP, as well as in the range and distribution of values. Certain groups, such as *Skilled Agricultural*,* Forestry*,* and Fishery Workers and Craft and Related Trades Workers* (SBP median: 140.0 mmHg [interquartile range: 130.0, 150.0]; DBP: 85.0 mmHg [80.0, 90.0]) and *Plant and Machine Operators and Assemblers* (SBP: 140.0 mmHg [130.0, 150.0]; DBP: 86.0 mmHg [80.0, 90.0]), exhibited higher median SBP and DBP values. The range of BP values varied within each occupational group, reflecting differing levels of variability among participants. Some groups, such as *Technicians and Associate Professionals*, *Service and Sales Workers*, and the *Unemployed*, showed a wider interquartile range, indicating greater variability in BP values. By contrast, BP in the first five occupational groups remained within a narrower and more consistent range, suggesting that occupational demands in these groups may be more comparable.

### Resting electrocardiogram outcomes

Analysis of the resting electrocardiograms revealed a sinus rhythm in 1,031 participants (99%). One participant (< 0.1%) had a pacemaker rhythm, while three (0.3%) showed atrial fibrillation at the time of recording, and five (0.5%) presented with another rhythm. A left anterior fascicular block was detected in 45 participants (4.3%). Estimates of the prevalence of left anterior fascicular block in the general adult population range from 1.0 to 2.5%, although the incidence increases with age [34, 35]. A complete left bundle branch block (LBBB) was present in eight participants (0.8%), and an incomplete LBBB in two (0.2%). The prevalence of LBBB also rises with age and is seen in less than 1% of the general population [36]. By contrast, a complete right bundle branch block (RBBB) was recorded in 20 participants (1.9%), and an incomplete RBBB in 78 (7.5%). Ventricular extrasystoles were found in 32 participants (3.1%), and supraventricular extrasystoles in 30 (2.9%). With the exception of incomplete RBBB, these abnormalities may suggest possible structural heart disease and should prompt further evaluation during initial diagnosis.

### Outcomes of clinical examination

The evaluation of the clinical examinations showed that findings such as abnormal heart murmurs, peripheral edema, wheezing or rhonchi in the lungs, and abdominal tenderness were generally unremarkable among participants. Abnormal heart murmurs were detected in 14 participants (1.3%), while peripheral edema was observed in 33 (3.2%). Additionally, 16 participants (1.5%) exhibited wheezing or rhonchi in the lungs, and 42 (4.1%) had abdominal tenderness.

### Blood analyses

**Fig. 3:**
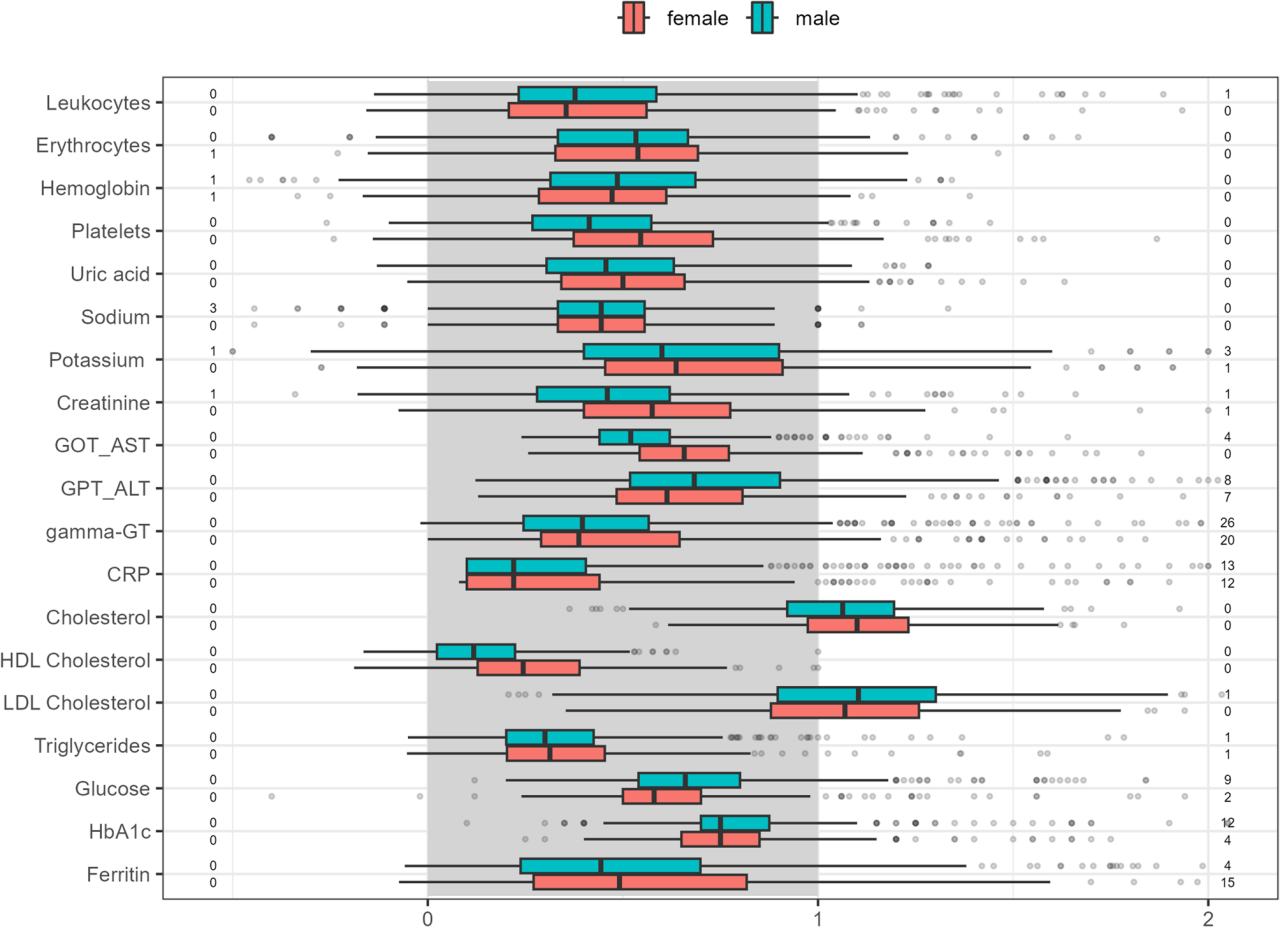
Boxplots of key blood analysis results (*n* = 1,033), shown in relation to reference values and stratified by sex. Red represents female participants, and blue represents male participants. The gray area indicates the reference range. The numbers on the left and right sides of each boxplot indicate how many values fall outside the range of [0.5, 2.0]

Figure3 presents the normalized blood analysis data of the participants, highlighting sex-related differences (red = female, blue = male). The gray area indicates the reference range, while the small numbers to the left and right of the x-axis show the number of outliers below 0.5 and above 2.0, respectively. These visuals illustrate the substantial variation in blood values across the sample. It should be noted that reference ranges differ for each parameter, and the boxplots reflect values scaled specifically to each parameter. Reference values are listed in Table S1 in the appendix.

Overall, the majority of participants had average blood values within the reference range for both sexes. However, elevated levels were observed for total cholesterol (mean 204.03 mg/dL [sd = 40.18], reference range < 190 mg/dL) and low-density lipoprotein (LDL) (mean 126.28 mg/dL [sd = 35.30], reference range < 116 mg/dL). Women showed higher median values than men for total cholesterol, while men had higher LDL values.

Other parameters, such as HbA1c and glutamate pyruvate transaminase, remained within the reference range but tended to be higher, particularly among male participants.

The blood values for the glomerular filtration rate, a key marker of kidney function, are not shown in the figure because of the absence of specific reference ranges in the laboratory analysis. Of the 1,033 participants, 623 (60%) had an estimated glomerular filtration rate of > 90 mL/min, which is considered within the range of normal renal function.

## Discussion

The variations in health parameters among different occupational groups underscore the influence of job-related physical activity on overall health. Sex-related differences are also evident across these parameters. Occupations involving physical labor, such as *Skilled Agricultural*,* Forestry*,* and Fishery Workers and Craft and Related Trades Workers*, tend to be associated with higher handgrip strength and muscle mass, particularly among males. By contrast, sedentary jobs are associated with higher BMI and PBF, especially among females (Fig. 1). As our sample included only individuals aged 45–59 years who were still in employment, a potential healthy worker effect should be considered; this group is likely healthier than the general population of the same age, which may partly explain the overall levels of functional measures such as handgrip strength.

Assessing physical activity levels at work remains challenging, even when using systems like ISCO-08. In 2017, a study by Deyaert et al. [37] sought to develop a method for assigning metabolic equivalent task values to different occupations using time-use data. The aim was to quantify physical activity across occupational groups and improve understanding of how occupational tasks influence overall health. Their findings revealed significant differences in physical activity levels between occupations, particularly in groups such as *Elementary Occupations* and *Craft and Related Trades Workers.*

Handgrip strength, in particular, shows substantial variation across occupational groups, but it should be regarded as an indicator of general muscle strength and functional capacity rather than a direct surrogate for occupational physical activity. Several published studies support this variation. For example, in 2020, Rostamzadeh et al. [38] compared (light) manual workers with office employees and found significantly higher handgrip strength among the (light) manual workers. This variation makes it difficult to draw broad, one-dimensional conclusions when discussing the relationship between handgrip strength and occupation. By contrast, a study in rural Ghana (by Dutch researchers) examined the association between handgrip strength and mortality, adjusting for factors such as age, sex, socioeconomic status, height, BMI, and drinking water source. The study found that only handgrip strength was significantly associated with mortality, specifically among Ghanaian manual workers, a group characterized by a high prevalence of malnutrition [39]. An Indian study found that handgrip strength varies depending on the type of manual tasks performed, such as cutting, pruning, digging, and spading, when using different hand tools. The study also identified differences based on the worker’s level of experience, categorizing participants into “beginner,” “intermediate,” and “advanced” groups [40]. Ultimately, grip strength was highest in the advanced group. While higher values in physically demanding occupations may plausibly reflect job-related muscular demands, elevated grip strength in certain high-income occupational groups could also be influenced by non-work factors such as leisure-time physical activity or supported by socioeconomic advantages.

Overall, the health status of employees over the age of 45 can be considered in need of improvement because a high demand for preventive measures was evident. When skill level is considered, non-academic professions, in particular, tend to fall outside the normal range in terms of body composition. The most common findings included elevated cardiovascular risk factors, such as obesity and high blood lipid levels. This is especially concerning because obesity is well established as one of the major risk factors in the development of cardiovascular disease, diabetes, and high BP [7]. According to the Robert Koch Institute, cardiovascular diseases are the leading cause of death in Germany, accounting for 40% of all deaths, despite being largely preventable through health-conscious behavior [41]. Preventive lifestyle changes can have a significant impact on key risk factors such as hypertension, obesity, diabetes mellitus, smoking, and physical inactivity [42–45].

These risk factors also include lipometabolic disorders, such as elevated levels of total cholesterol and LDL, as well as reduced levels of high-density lipoprotein. As shown in Fig. 3, participants in this study exhibited elevated cholesterol and LDL values in both sexes, while high-density lipoprotein values were in the lower end of the normal range. These findings align with those of the German Health Interview and Examination Survey for Adults (DEGS1), which reported that 56.6% of men and 60.5% of women aged 18 to 79 years had elevated total cholesterol levels (≥ 190 mg/dL). When the DEGS1 results are considered alongside the findings of this study, the potential benefits of implementing a nationwide preventive health check-up become clear. In DEGS1, elevated total cholesterol levels were not only identified through blood analyses, but participants were also asked about prior medical diagnoses of lipometabolic disorders. The evaluation revealed that approximately two-thirds had a lipid metabolism disorder, and more than half of them were unaware of it [46]. According to the World Health Organization [47], elevated cholesterol is one of the major risk factors for heart disease and stroke, accounting for one-third of ischemic heart disease cases worldwide, in both developing and industrialized countries. Early detection and subsequent treatment, such as lifestyle changes or drug therapy, could help reduce or prevent these serious secondary diseases [47].

When examining BMI and PBF in relation to occupational groups, a Finnish-Japanese study highlights a clear socioeconomic gradient in obesity within the Finnish population, a trend not observed in the Japanese population. Although both countries are industrialized, factors such as differences in traditional diets and varying obesogenic environments likely contribute to these disparities [48]. In the present study, a similar pattern can be seen in the PBF data for female participants, with an increase in body fat percentage across occupational groups from *Managers* to the *Unemployed*, mirroring the trend observed in the Finnish cohort. However, this trend is less apparent among male participants. A similar observation applies to BMI across occupational groups, for both men and women. These findings underscore the need for targeted health interventions and preventive strategies that are tailored to the specific needs of different occupational groups and sexes.

Furthermore, given the high risk of cardiovascular diseases, it is notable that 40 of 100,000 residents die of sudden cardiac arrest annually [49]. In our study, the majority of participants showed no signs of cardiological abnormalities. The prevalence of LBBB was 0.8% (*n* = 8) for complete LBBB and 0.2% (*n* = 2) for incomplete LBBB. The literature indicates that LBBB prevalence increases with age, as demonstrated in a longitudinal study of 855 Swedish men born in 1923 and followed for 30 years beginning at the age of 50 years [36]. In that cohort, the prevalence of LBBB was 0.4% at age 50 years, rising to 2.3% at age 75 years and 5.7% by age 80 years, suggesting that LBBB may reflect a slowly progressive, degenerative disease of the cardiac conduction system [50]. By contrast, other studies associate new-onset LBBB with advanced heart disease, particularly in older adults [51]. When LBBB is detected, patients should be further evaluated for potential underlying conditions such as hypertension, coronary artery disease, myocarditis, valvular heart disease, and cardiomyopathies.

RBBB was observed in 1.9% (*n* = 20) of participants in our study, with an additional 7.5% (*n* = 78) presenting with incomplete RBBB. Previous studies confirm that the prevalence of RBBB increases with age. One prospective study reported an increase from 0.8% at the age of 50 years to 11.3% by the age of 80 years [36]. Similarly, the NHANES study found an RBBB prevalence of 2.3% among participants with a mean age of 61 years [52]. Incomplete RBBB, defined by a QRS duration between 100 and 119 ms, can occur in healthy individuals and was observed in 3.4% of participants in the Copenhagen City Heart Study, with no significant correlation to age [53].

A comprehensive diagnostic approach is recommended for detecting cardiological abnormalities. Furthermore, both the European Society of Cardiology and the German Society of Cardiology emphasize the need for valid preventive assessments and risk factor evaluations, such as those using the SCORE algorithm [54], as essential tools in managing individual health. These recommendations underline the importance of regular health check-ups, which in the German context are commonly offered as part of workplace health promotion or through statutory health insurance, though participation is voluntary. Given the high prevalence of elevated BMI, PBF, and lipid abnormalities in our sample, particularly in certain occupational groups, such preventive examinations could help to identify at-risk individuals earlier and enable targeted interventions before chronic diseases develop.

There is ongoing debate regarding the cost–benefit ratio and effectiveness of preventive health check-ups. Some studies have found no clear association between preventive check-ups and reduced mortality or a lower incidence of cardiovascular disease [55]. However, other research has linked preventive health examinations to improvements in health behavior and quality of life [18]. Even if these check-ups do not demonstrably reduce mortality risk, the resulting improvement in quality of life appears to justify their implementation, particularly given studies that show strong associations between quality of life, well-being, and the ability to work. For example, a study by Tavakoli-Fard and Mortazavi [56] showed that health-related quality of life is a key factor in women’s ability to work, a connection that seems likely to apply to men as well. Chang and Yeh [57] also demonstrated that work ability significantly influences quality of life in individuals with musculoskeletal disorders, suggesting that the relationship may be reciprocal. Additionally, Liss and Uchida [18] showed that screening contributes to the early detection of chronic conditions such as depression and high blood pressure, helps identify risk factors, and increases the uptake of other preventive health services.

Examples from the German context illustrate the potential of workplace health promotion programs. In social firms and other workplace settings, multi-component interventions combining physical activity promotion, dietary counseling, and smoking cessation support have been implemented and shown to positively influence employees’ health parameters, including BMI, blood pressure, and physical fitness [58]. A recent systematic review of workplace health promotion programs in industrial sectors further confirmed that such integrated approaches can lead to measurable improvements in health outcomes, although effects vary by intervention design and target group [59]. These programs often benefit from the established structures of occupational health services and statutory health insurance in Germany, making them more accessible compared to contexts where healthcare access depends on individual insurance coverage. In addition to the previously mentioned benefits of preventive health check-ups, the financial burden on the healthcare system, particularly due to overweight and obesity, should also be taken into account. In Germany, these costs are estimated to range between 22.2 and 23.0 billion euros [60]. Comprehensive preventive measures could play a significant role in helping to reduce these expenses.

Another strong argument for general health check-ups is the opportunity for initial diagnoses through comprehensive clinical examinations. Many symptoms may go unnoticed or be ignored by patients. As such, a general check-up combined with a thorough medical consultation may be an essential component in managing an individual’s healthcare. Moreover, physician guidance, especially when delivered through an interactive dialogue, can have a lasting, positive influence on an individual’s support system for maintaining a healthy lifestyle.

From a workplace perspective, the job demand–control model offers one possible framework to interpret some of the occupational differences observed in our study. While we did not directly assess psychosocial work factors, higher occupational status in our sample was associated with lower BMI and BP, which aligns with literature suggesting that greater job control is linked to healthier lifestyle behaviors. In 2021, Tipayamongkholgul et al. [61] found that unskilled workers are at higher risk for noncommunicable diseases such as cardiovascular disease, diabetes, and hypertension. As noted, greater job control can foster motivation for physical activity, reducing the risk of both psychological and physical conditions [62, 63]. These findings support the outcomes observed in our study regarding the health status of specific occupational groups.

Another model that addresses the workplace is the gratification crisis model. According to this theory, a well-balanced relationship between demands and rewards is essential to prevent psychological conditions such as burnout or exhaustion [64]. This underscores the significant impact that workplace atmosphere and conditions can have on both an employee’s well-being and overall life. While our study did not include specific data on workplace conditions or job motivation, we recommend that future research incorporate these factors. Doing so would offer a broader perspective when assessing participants’ risk factors related to workability and health.

Ultimately, the results strongly suggest that health awareness among the 45- to 59-year-old population should be improved. Targeted educational efforts could help reduce risk factors and prevent potential illnesses, thereby supporting both workability and quality of life into older age. The implementation of a nationwide preventive health check-up appears to be a promising approach.

In response to the research questions, the general health status of the sample indicates considerable health concerns, including obesity, elevated cardiovascular risk, and high blood lipid levels. The average BMI in the sample was 26.89 kg/m^2^, with clear occupational and sex-related differences in anthropometric measures and cardiovascular risk factors. Specific occupational groups displayed distinct health profiles; for instance, *Plant and Machine Operators and Assemblers* had the highest mean BMI (28.25 kg/m^2^), while individuals in *Elementary Occupations* exhibited the highest average PBF (32.53%). These differences highlight the importance of tailored occupational health interventions that account for the unique demands and risks associated with each profession.

In summary, our findings directly address the three major research gaps identified in the Introduction: (I) we combined objectively measured clinical health indicators with standardized self-reported health information, overcoming the limitations of studies based solely on self-report; (II) we classified occupations according to the internationally recognized ISCO-08 system, thereby ensuring international comparability; and (III) we provided recent, occupation-stratified health profiles of midlife employees in Germany, which had been lacking to date. By explicitly closing these gaps, our study offers an important contribution to both national and international occupational health research and provides a robust foundation for designing targeted, evidence-based prevention strategies.

### Strengths

One of the primary strengths of this study is its substantial sample size (*n* = 1,040), which provides a robust dataset for comprehensive statistical analyses. The minimal amount of missing data further enhances the reliability and validity of the findings, allowing for more precise interpretations and conclusions.

Moreover, the survey methods used in the Ü45-Check ensure validated results and could be applied in general medical practice [65]. A particular methodological strength is the combination of objectively measured clinical health indicators, including anthropometrics, blood pressure, lipid panels, and handgrip strength assessed by trained physicians, with standardized self-reported health information. This approach reduces bias associated with self-reported data alone and allows for a more precise characterization of health status. In addition to standardized techniques for assessing body composition, validated tools like handgrip strength measurement were also employed [66]. Handgrip strength is gaining importance as a screening tool, as reduced SMM is associated with lower quality of life and impaired physical function [30]. Handgrip strength is therefore an indicator of muscle function, especially for older adults [31]. In this study, it was applied as a general marker of muscle strength rather than a direct surrogate for workplace physical activity.

Another strength is the structured classification of participants’ jobs according to ISCO-08, enabling systematic occupational comparisons and direct national and international data comparability while addressing key methodological gaps. While this approach offers valuable insight into how occupational demands and environments affect health, substantial variation in job tasks can still exist within each major ISCO-08 group.

Early identification of health risk factors is essential for preventing or mitigating disease and improving overall health outcomes [67–69]. Individuals benefit from the early detection of such risks, which also positively influences quality of life [7–9]. In addition to individual benefits, early detection has the potential to reduce healthcare costs by preventing non-communicable diseases like obesity, which place a significant financial burden on social security systems [9, 17, 65].

Finally, this study highlights the importance of occupational and sex-specific health interventions. The data reveal clear differences in health profiles across occupational groups and between men and women, emphasizing the need for targeted, tailored public health strategies.

## Limitations

The limitations of this study include the limited generalization of the results, as it cannot be guaranteed that the sample examined represents the average of the population. This is partly due to the specific recruitment process and the population subset targeted. For example, language barriers or a lack of education may result in an inadequate understanding of the invitation letter to participate in the study, or might not be considered as important. Another reason for the lack of transferability of the results to the overall population is how the participants were selected. Since the participants were contacted by the German Pension Fund, certain occupational groups in Germany, such as civil servants, who are not insured by the German Pension Fund, could not be included in the sample. Moreover, within the included ISCO-08 major groups, considerable diversity in actual job tasks and exposures remains, which limits the ability to draw conclusions for all occupations within a given group.

In addition, a possible bias could be that people who are already more health-conscious may have seen participation in the study as more relevant and worthwhile. Certain recorded parameters, such as hours of physical activity per week, the level of activity at work, etc., were determined by subjective assessment. Here, too, a bias may have caused distortions and over- or underestimations.

## Conclusion

This analysis offers a comprehensive overview of the health status of employees aged 45 to 59 years in Germany across various occupational groups. The findings reveal disparities in anthropometric and clinical measures, influenced by occupational demands and sex differences. The results clearly indicate that the general health status of this population requires improvement, with obesity and elevated cardiovascular risk being particularly prevalent. By analyzing key health indicators, this study highlights the need for occupational health interventions tailored to the specific challenges and risks associated with different job types and genders. A nationwide preventive health check-up emerges as a valuable strategy for identifying and addressing health risk factors among employees at an early stage.

## Supplementary Information

Supplementary Material 1.

## Data Availability

No datasets were generated or analysed during the current study.

## Abbreviations

BMI: Body mass index
BP: Blood Pressure
DBP: Diastolic Blood Pressure
DEGS1: German Health Interview and Examination Survey for Adults
ISCO-08: International Standard Classification of Occupations 2008
LBBB: Left Bundle Branch Block
LDL: Low-density Lipoprotein
PBF: Percent Body Fat
RBBB: Right Bundle Branch Block
SMM: Skeletal Muscle Mass
SBP: Systolic Blood Pressure
sd: standard deviation
Ü45: people over 45 years old
