# Supplementary material for: The general health status of employees in Germany aged 45 to 59 years (Ü45-Check) – a cross-sectional study
Source: BMC Public Health. 2025 Sep 24;25:3051. doi: 10.1186/s12889-025-24778-7 (PMC12459031; doi:10.1186/s12889-025-24778-7)
Supplement: Supplementary file 1 — Supplementary Material 1. [file 12889_2025_24778_MOESM1_ESM.docx]

| **Table S1** Reference ranges of blood values for male and female | | | |
| --- | --- | --- | --- |
| **Blood Value** | **Unit** | **Reference Range** | |
|  |  | ***male*** | ***female*** |
| Leukocytes | /nl | 3.90-10.50 | 3.90-10.50 |
| Erythrocytes | /pl | 4.3-5.8 | 3.9-5.2 |
| Hemoglobin | g/dl | 13.5-17.0 | 12.0-15.6 |
| Hematocrit | l/l | 0.395-0.505 | 0.355-0.455 |
| MCV | fl | 80.0-99.0 | 80.0-99.0 |
| MCH | pg | 27.0-33.5 | 27.0-33.5 |
| MCHC | g/dl | 31.5-36.0 | 31.5-36.0 |
| RDW-CV | % | 11.5-15.0 | 11.5-15.0 |
| Platelets | /nl | 150-370 | 150-370 |
| MPV | fl | 7.0-12.0 | 7.0-12.0 |
| Neutrophils absolute | /nl | 1.50-7.70 | 1.50-7.70 |
| Immature granulocytes absolute | /nl | < 0.050 | < 0.050 |
| Lymphocytes absolute | /nl | 1.10-4.50 | 1.10-4.50 |
| Monocytes absolute | /nl | 0.10-0.90 | 0.10-0.90 |
| Eosinophils absolute | /nl | 0.02-0.50 | 0.02-0.50 |
| Basophils absolute | /nl | 0.00-0.20 | 0.00-0.20 |
| Neutrophils % | % | 42.0-77.0 | 42.0-77.0 |
| Immature granulocytes % | % | 0.0-1.0 | 0.0-1.0 |
| Lymphocytes % | % | 20.0-44.0 | 20.0-44.0 |
| Monocytes % | % | 2.0-9.5 | 2.0-9.5 |
| Eosinophils % | % | 0.5-5.5 | 0.5-5.5 |
| Basophils % | % | 0.0-1.8 | 0.0-1.8 |
| Sodium | mmol/l | 136-145 | 136-145 |
| Potassium | mmol/l | 3.5-4.5 | 3.4-4.5 |
| Iron | µmol/l | 5.8-34.5 | 5.8-34.5 |
| Magnesium | mmol/l | 0.66-1.07 | 0.66-1.07 |
| Urea | mg/dl | 17-48 | 17-48 |
| Creatinine | mg/dl | 0.70-1.20 | 0.50-0.90 |
| Estimated GFR |  |  |  |
| Creatine kinase | U/l | < 190 | < 167 |
| GOT | U/l | < 50 | < 35 |
| GPT | U/l | < 41 | < 31 |
| Gamma-GT | U/l | 8-61 | 5-36 |
| Uric acid | mg/dl | 3.6-8.2 | 2.3-6.1 |
| CRP | mg/l | < 5.0 | < 5.0 |
| Total cholesterol | mg/dl | < 190 | < 190 |
| HDL cholesterol | mg/dl | > 40 | > 45 |
| Non-HDL cholesterol | mg/dl |  |  |
| LDL cholesterol | mg/dl | < 116 | < 116 |
| Triglycerides | mg/dl | < 150 | < 150 |
| Glucose in fluoride | mg/dl | 60-110 | 60-110 |
| HbA1c | % | < 6.0 | < 6.0 |
| HbA1c mmol/mol | mmol/mol | < 42.0 | < 42.0 |
| TSH basal | mU/l | 0.27-4.20 | 0.27-4.20 |
| Ferritin | µg/l | 30.0-400.0 | 13.0-150.0 |
| *Notes.* CRP C-reactive Protein; GFR Glomerular Filtration Rate; Gamma-GT Gamma-Glutamyl Transferase; GOT Glutamate Oxaloacetate Transaminase; GPT Glutamate Pyruvate Transaminase; HbA1c Glycated Hemoglobin; HDL High-Density Lipoprotein; LDL Low-Density Lipoprotein; MCH Mean Corpuscular Hemoglobin; MCHC Mean Corpuscular Hemoglobin Concentration; MCV Mean Corpuscular Volume; MPV Mean Platelet Volume; RDW-CV Red Cell Distribution Width; TSH Thyroid Stimulating Hormone | | | |
